# Supplementary material for: Are carbon emissions trading and green financial instruments synergistic? -Comprehensive quantitative research based on content analysis
Source: PLoS One. 2024 Mar 7;19(3):e0298601. doi: 10.1371/journal.pone.0298601 (PMC10919601; doi:10.1371/journal.pone.0298601)
Supplement: S1 Data — (ZIP) [file pone.0298601.s001.zip › Data/地级市Z_F工作报告.zip!/╣π╢1⁄2/╒┐╜¡╩╨/└┤╫╘╣1⁄2╓┌║┼í╛┬φ┐╦╩2╛▌═°í┐_2016.docx]

各位代表：

　　现在，我代表市人民政府向大会报告政府工作，请予审议，并请市政协各位委员和其他列席人员提出意见。

　　一、2015年及“十二五”时期工作回顾

　　2015年是“十二五”收官之年，也是湛江经受考验、克服困难、收获硕果的奋进之年。一年来，在市委坚强领导下，在市人大、市政协监督支持下，我们认真贯彻党的十八大和十八届三中、四中、五中全会精神，深入贯彻习近平总书记系列重要讲话精神，认真实施省委、省政府促进粤东西北振兴发展战略，认真落实市委十届七次、八次全会精神，紧紧依靠全市人民共克时艰、稳中求进，积极应对重大自然灾害和经济下行压力，有效保持经济社会平稳健康发展，较好完成市第十三届人大五次会议确定的各项任务，较好完成“十二五”规划主要目标任务，为“十三五”全面建成小康社会打下了坚实基础。

　　过去一年，全市经济运行总体平稳、稳中有进。完成生产总值2380亿元，增长8.5%。规上工业总产值2259.6亿元、增长9.8%，增加值698.8亿元、增长9.9%。服务业增加值1019.6亿元，增长9.1%。固定资产投资1313.7亿元，增长28.7%。社会消费品零售总额1298亿元，增长11.7%。来源于湛江的财政总收入491.6亿元，公共财政预算收入121.9亿元、增长2.7%。居民人均可支配收入16631.7元，增长8.7%。年末金融机构存款余额2675亿元，增长10.5%，存贷比58.4%。全社会用电量116.04亿千瓦时，增长6%。钢铁基地1号高炉点火投产。省第十四届运动会暨第七届残运会成功举办，2015中国海博会、水博会精彩升级，第三届湛江海洋周和第十五届中国网络媒体论坛圆满成功。

　　过去一年，我们先后遭遇历史罕见的干旱和台风灾害。上半年，雷州半岛出现有气象记录以来降雨量最少、高温天数最多的严重旱情，全市170万亩农作物、183万群众受灾。下半年，遭遇建国以来秋季登陆我国大陆的最强台风“彩虹”正面袭击，中心城区大面积停电停水，交通、通讯受阻中断，城市功能近乎瘫痪，受灾人口345.5万、企业1230家，直接经济损失256.5亿元。大灾面前，我们在省委、省政府坚强领导下，团结带领全市人民奋力防灾救灾，尤其是有效处置钢铁基地断电、富多煤气球罐泄漏等重大险情，有序恢复城市秩序，恢复企业生产，完成“全倒户”重建，取得了抢险救灾、重建家园的全面胜利！

　　一年来，我们主要做了以下工作：

　　（一）推进改革开放，发展活力有效释放。改革取得新突破。基本完成改革任务237项；发布市级行政审批事项目录，市级审批事项取消28项、下放11项。市文化中心等23个项目纳入省PPP项目库，“南方海谷”、湛化环保搬迁等9个项目获得国家8.1亿元贴息贷款基金支持。出台23条扶持创业创新政策，新增个体工商户35098户、增长9.8%，私营企业5149家、增长16.7%；新增大学生创业主体668户、投资人872人，增幅超过4倍；新增省级众创空间1家。19家中小微企业在沪深股交中心挂牌，中小微企业“互保金”入池企业110家。民间投资788.3亿元，增长30%；民营经济增加值1526.3亿元，增长9.7%。助农取款服务实现行政村全覆盖，粤西首家惠民村镇银行开业。县镇医疗卫生服务管理一体化改革全面实施。基本完成机关事业单位工资、市县两级公务用车和事业单位分类改革。市区环卫作业走向市场化。组建公共资源交易中心。创新驱动发展成效明显。“南方海谷”创新科技园动工建设，设立首支“南方海谷”股权投资基金，首期认缴基金1.58亿元。成功举办首届“南方海谷杯”海洋科技创新创业和“市长杯”工业设计大赛。专利授权2486件，增长92.1%，增幅居全省第一。新增7家省级工程中心和1家院士工作站，新认定国家高新技术企业5家，新增省高新技术入库培育企业40家。对外开放迈出新步伐。2015中国海博会参展国家、企业和专业观众增幅超过30%，成为中国十大特色展会之一。第二届中国水博会交易额180亿元，增长20%。保税物流中心建成并通过预验收。湛江港获准扩大开放，徐闻港、吴川进出境货运车辆检查场和雷州乌石对台小额贸易口岸获准对外开放。粤西首个国际进出境快件监管中心验收运营。市政府驻俄罗斯、东盟（泰国）经贸代表处挂牌运作。外贸进出口总额51.5亿美元，实际利用外资1.57亿美元。“三地”融合发展成效初显。奋勇高新区成为全省唯一的国家级军民结合产业示范基地。24所高校向我市94家企业派驻科技特派员152人次。垦地共建国家现代农业示范区广前核心区启动实施。

　　（二）推进结构调整，产业建设迈向新起点。工业发展有历史性突破。155个重点项目完成投资452.3亿元，其中92个省级重点项目完成投资383.8亿元，居粤东西北地区首位。112个工业项目投产，35个项目完成技术改造，新增“个转企”286户、“企转规”60户。钢铁基地完成投资179亿元，累计完成投资380亿元，十大主体工程建设全面加快，自备电厂并网发电，两个矿石配套码头建成运行，2918名产业工人持证上岗。中科炼化具备动工条件。晨鸣纸业三期投产、四期动工，实现工业产值125亿元，增长31.6%。中海油重交沥青、华德力电气、国投生物能源燃料乙醇、丰诚水泥、一品木业等项目建成投产，恒诚中药、南国药业完成迁建，珠江啤酒、圣华玻璃、湛江卷烟厂等企业通过技改扩能增效，大唐雷州火电、京信东海电厂和鹏尊能源动工。落实22条受灾企业复产扶持办法，市财政贷款贴息1亿元，412家停产企业恢复生产。淘汰5家落后产能企业。产业园区新开工项目44个、建成投产30个，规上工业总产值突破1000亿元。湛江开发区新上钢铁配套项目78个，进入“中国产业园区持续发展100强”。奋勇高新区11个项目建成投产。廉江产转园连续四年省级考核优秀。

　　服务业迈上新台阶。引入阿里巴巴、京东等著名电商，南菜北运电子交易平台和绿源果菜物联网建成上线，软件与信息消费增长20%，进入“2015中国大陆最佳商业城市排行榜”。新增集装箱班轮航线7条，港口吞吐量2.2亿吨，增长8.9%，增幅居全国沿海港口第三。民航首开湛江至曼谷国际航线，机场旅客吞吐量120.7万人次，增长20%。被评为“2015中国十大避寒旅游名城”，南三岛成为“中国十大美丽海岛”。旅游接待3300万人次，增长14%；总收入271亿元，增长34.6%。商品房销售额180.4亿元，增长7.6%；销售面积325.3万平方米，增长10.6%。住房公积金用于住房消费资金47.9亿元，增长36.7%。

　　现代农业持续发展。完成农业总产值721.1亿元，增长3.7%。启动编制现代农业、水利设施、生态修复三大规划。新增市级以上农业龙头企业15家、农民合作社419家。建立农村经济联合社1676个，建成农村产权流转管理服务平台97个，完成流转交易额6441.4万元。建成22个省级菜篮子基地，正大（湛江）集团祖代种猪场投产。建成高标准基本农田135.4万亩。政策性农村住房保险全覆盖，水稻参保覆盖面92.2%，甘蔗、玉米、香蕉参保面积分别达到67.2万亩、3.14万亩和3390亩。在全省率先开展“农技宝”试点。新造200吨以上大型钢质渔船72艘，远洋渔业规模居全省第二。实施水利工程230宗，完成投资19.3亿元。雷州青年运河节水改造基本完成。

　　（三）推进协调发展，城乡建设迈出坚实步伐。交通基础设施建设力度加大。完成交通建设投资122.9亿元，增长80%。东海岛疏港一级公路通车，建成广湛高速官渡出入口立交。云湛高速湛江段、东海岛铁路全线施工，东雷高速、湛徐高速徐闻港支线动工建设。改造国省道550公里、县乡道290公里，硬化村道840公里。湛江国际机场迁建列入国家、省“十三五”及民航发展专项规划，项目选址获批。完成湛江港30万吨级航道维护，霞山港区30万吨级散货码头主体工程投入试运行。鉴江水利枢纽向钢铁基地供水。完成电网投资23.3亿元，东海岛500千伏输变电工程提前运行。

　　城市扩容提质有新进步。获得全国文明城市提名资格，进入中国智慧城市百强榜。奥体中心“一场三馆”和水上运动中心、民大喜来登酒店成为城市新地标。海东新区总体规划获省批准，24个项目建成。17公里海东快线北段建成通车，钢铁基地住宅小区一期基本建成，广东医学院附属医院海东院区、南粤银行金融大厦等项目加快推进，海东体育休闲公园建成开放，完成那面村搬迁和三柏西村拆迁。主城区建成新湖大道、振兴路和广州湾大道中澳友谊花园段等7条市政道路；完成海湾路、寸金路和海光路“白改黑”，完成三帆环岛路口、绿华路与海滨大道交叉路口改造，完成“三旧”改造面积1244亩，中央商务区、城市原点广场、渔人码头、万达广场等项目加快推进。市交警智能指挥中心启用，市数字化城市管理中心建成，城乡光缆基本覆盖。确定高铁西客站建设方案，西城新区规划通过市政府审批。

　　城市转型成效显现。“三个城市”建设获得社会支持。制定脚印城市建设规划技术导则，开展中心城区TOD、慢行系统、公共交通体系等规划修编；32条市区干道路权向公交车、自行车和行人倾斜；新辟公交专用道20多公里，新增及更新公交车390辆；建成公共自行车租赁点472个，投放自行车1万辆，日均租车超过2.6万人次；清除人行道“拦路虎”300多个，拆除违法建筑11.4万平方米；市区建成绿道10公里，瑞云湖、滨湖公园和市区主干道慢行景观廊道基本形成。南三岛东郊10公里绿道动工。出台海绵城市建设方案，实施主城区治水十年规划和三年计划，完成8条河涌清淤截污和6个“水浸街”黑点整治。成功申报建设国家循环经济示范城市，湛江开发区成为国家级循环化改造示范试点园区。

　　县域经济与县城建设有新亮点。深入开展“三讲三评”，县（市、区）工业发展持续升温，廉江市生产总值突破400亿元。碧桂园等著名地产企业落户雷州、廉江城区。雷州城区启动建设8.8公里环西湖水库绿道。吴川城区完成沿江路二期、新建路和长寿路改造。廉江城区完成11条干道“白改黑”，通过道路拓展与“三旧”改造，城市规模扩大、品位提升，成为县城建设新标杆。

　　环境保护有效加强。坚守发展底线，推进节能减排，完成省下达单位GDP能耗降低2.33%的目标任务。平乐再生水厂投入运行，赤坎水质净化厂三期动工，小东江环境污染综合整治初见成效。生活垃圾焚烧发电厂、医疗废物处理厂建成，晨鸣纸业碱回收处理厂投入使用。农村生活垃圾转运“一镇一站”、“一村一点”投入运营，80%以上生活垃圾得到有效处理，农村保洁实现全覆盖。关停4个重污染结构减排项目，取缔72家污染企业，淘汰黄标车1.48万辆、高耗能电机17万千瓦、燃煤锅炉51台，PM2.5年均值从上年每立方米29微克降为28微克。城市饮用水源、近岸海域功能区水质达标率100%。更新造林20.5万亩，营建生态公益林4万亩，种植沿海防护林两万亩，绿化美化乡村212条。

　　（四）加大民生投入，百姓福祉持续增进。市财政在增收放缓情况下，社会民生投入336.3亿元，增长61.2%，占总支出80.4%。十大民心工程加快推进，十件民生实事全面完成。

　　民生保障有效提升。新增城镇就业7.76万人，失业人员再就业3.78万人，农村劳动力转移就业6.15万人。企业退休人员基本养老金人均每月增至2090元，失业保险金标准每月增至968元。城乡居民医保参保率98.8%，儿童急性白血病等6种疾病纳入重大疾病保障范围，职工医保特定门诊病种扩大到26种。城乡居民低保标准人均每月分别提高到410元和260元，孤儿集中与分散供养低保标准人均每月分别提高到1240元和760元。80周岁以上高龄老人全面享受普惠型津贴，养老服务中心主体工程基本建成。开工建设保障房7601套、建成3041套；动工改造茅草房15227户、竣工12174户，建成移民新村85条；钢铁基地1.2万搬迁村民全部搬进新居；1561户因灾“全倒户”新房竣工；基本解决68个社区办公用房不足和400条自然村、50万农村群众饮水安全问题。实施扶贫项目32.2万个，250条村3.2万户、16.38万贫困人口稳定脱贫。

　　社会事业持续进步。城乡免费义务教育生均公用经费补助标准小学、初中分别提高200元和400元；改革普通高中招生制度，推行指标到校，取消择校生，促进教育公平发展；10个县（市、区）、121个镇（街）通过教育强县、强镇验收，成功创建广东教育强市；8个县（市、区）通过国家义务教育发展基本均衡县评估认定；完成18所薄弱普通高中改造提升，建成8所县级特殊教育学校。湛江中心人民医院新院和卫校新校区加快建设；成功防控埃博拉和中东呼吸综合征等疫情。进入“中国文化城市100强”，启动百部地方志丛书修编，雷剧、吴川南派粤剧成功申报国家非物质文化遗产，廉江舞鹰雄艺术获中国民间艺术“山花奖”。参加省第十四届运动会、第七届残运会分获团体总分第五名和第二名。“明厨亮灶”、“掌上FDA”等食品药品安全监管经验在全省推广。海关、检验检疫、海事、边检等口岸单位深化“三地七方”合作，落实“信息互换、监管互认、执法互助”，推进“单一窗口”建设，有效支持地方开放发展。审计、统计、粮食、质监、民族宗教、老区、应急、海防、人防、气象、残疾人、地震、港澳、外事侨务、妇女儿童等工作取得新成绩。

　　民主法制建设不断推进。成为全省首批获得地方立法权的设区市。平安建设全面推进，社会大局保持稳定。加强安全生产监管，连续7年未发生死亡10人以上重特大事故。市区新装一类视频监控探头1600个，建成治安卡点41个。纵深开展“3+2”专项打击行动，市区街面“两抢”警情下降26.6%。“六五”普法通过验收。查获走私及处理无主货物案件74宗。推行责任、阳光、法治信访，信访秩序明显好转。

　　（五）践行“三严三实”，政府建设进一步加强。扎实开展“三严三实”专题和“守纪律讲规矩做表率”主题教育，坚持从严从实改进政府工作。依法行政考评等次良好。主动接受人大依法监督和政协民主监督，办理人大代表建议、政协委员提案257件，办复率100%。认真履行“一岗双责”，政府系统党风廉政建设进一步加强。全面落实省委第七巡视组反馈意见，完善规章制度，扎实抓好工程建设领域整改工作。严格执行中央八项规定，驰而不息纠正“四风”，“三公”经费、会议费支出下降14.6%。市政府行政服务中心实体和网上办事大厅功能不断完善，坚持市直部门副处以上干部值守12345市民服务热线平台制度，市民诉求办结率96.9%。举办市政府新闻发布会12场、在线访谈18期，市政府门户网站获得2015中国地级市政府网站绩效评估、省政府网站公共服务程度测评第六名。

　　各位代表，一年来令人振奋的发展成效，标志着湛江走过辉煌“十二五”。“十二五”期间，我们在省委、省政府和市委坚强领导下，历经考验，砥砺奋进，一步一个脚印，一年一个台阶，走过了不平凡的发展征程。

　　五年来，湛江综合实力稳步提升，实现了发展之变：生产总值年均增长10.6%，实现了从1000亿元向2000亿元的跨越；第三产业增加值突破1000亿元。公共财政预算收入年均增长13％。固定资产投资实现翻番，累计突破4000亿元，年均增长28％。产业结构持续优化，实现了工业之变：钢铁基地、晨鸣纸业等重大项目建成投产，3家企业年销售收入和6个行业年产值超100亿元，工业结构有效改善，主导地位全面突显。港口运输、商贸物流、滨海旅游等现代服务业加快发展。粮食、蔬菜和热带作物产量居全省首位。人居环境有效改善，实现了城市之变：海东开发规模初具，海西提质成效明显，港湾清障圆满完成，“一湾两岸”生态型海湾城市格局基本形成；一批公园绿地和污水、垃圾处理设施建成使用，有效开展九洲江流域和市区河渠整治，环境质量保持优良，成为国家卫生城市。主动融入世界，实现了开放之变：中国海博会、水博会永久落户、成功举办，保税物流中心建成，对接东盟合作发展势头良好，成为首批“一带一路”海上合作战略支点城市。财政投入力度加大，实现了惠民之变：社会民生投入超过财政总支出的80%，教育、文化、卫生、体育等事业全面发展，圆满完成扶贫开发“双到”任务。

　　各位代表，经过“十二五”的奋斗磨练，湛江经济更加繁荣，城市更加美丽，社会更加进步。这是市委正确领导的结果，是市人大、市政协有效监督的结果，是驻湛部队、武警官兵、公安干警使命必达的结果，是中央、省驻湛单位、各民主党派、人民团体以及社会各界人士团结协作的结果，是省直部门大力支持、兄弟城市无私援助的结果，更是全市广大工人、农民、知识分子、干部职工奋发有为、顽强拼搏的结果。我谨代表市人民政府，向所有关心和支持湛江发展的同志们、朋友们，表示衷心的感谢，致以崇高的敬意！

　　“十二五”发展成就来之不易，经验弥足珍贵。回顾五年来的工作，我们深切体会到：实现湛江振兴，必须坚持信念在心，抓紧发展第一要务，胸怀理想、点燃激情、主动作为；坚持责任在肩，推进实施“工业立市、港口兴市、生态建市”发展战略，坚守“既要经济崛起、又要蓝天碧水”，坚定创新、协调、绿色、开放、共享发展；坚持机遇在我，主动适应新常态，厚植发展新优势，全力稳增长、调结构、促改革、惠民生、防风险；坚持实干在先，大干快干，攻坚克难，砥砺前行，以行动落实，用结果说话，创造无愧于时代的业绩；坚持宗旨在民，同心同德，众志成城，凝聚改革发展的强大合力，让人民拥有更多获得感；坚持法纪在前，遵纪守法，转变作风，确保干部清正、政府清廉、政治清明，营造风清气正的良好政治生态和干事创业氛围。

　　过去五年，成绩固然可喜，问题必须正视。主要体现在：经济总量小，人均水平低；部分重大项目推进缓慢；传统产业升级、新兴产业成长不快，经济下行压力还在加大；对外交通条件亟待改善；港口竞争力有待提升；县域经济发展不充分、不平衡；社会民生欠账较多等等。对于这些问题，我们必须付出更大努力加以解决。

　　二、“十三五”时期奋斗目标和主要任务

　　根据中共湛江市委十届八次全体会议和市委《关于制定国民经济和社会发展第十三个五年规划的建议》精神，市政府编制了《湛江市国民经济和社会发展第十三个五年规划纲要》（草案），提请大会审议。

　　决战“十三五”，全面奔小康，我们拥有宝贵的战略机遇，面临严峻的困难挑战。未来五年，全球经济在深度调整中艰难复苏，国内经济长期向好的基本面没有改变，湛江依然处在大有作为、振兴发展的重要战略机遇期。湛江的机遇，在于国家建设海洋强国和制造强国，实施军民融合发展战略；在于国家推动共建“一带一路”，支持湛江建设海上合作战略支点城市；在于省委、省政府支持湛江建设环北部湾中心城市；在于湛江振兴发展的内生动力正在形成。当前和今后一段时期，湛江经济运行稳中有进、稳中向好的发展趋势不变，特别是临港工业持续发展的趋势不变，新型城镇化向前推进的趋势不变，社会“众创”热潮涌动的趋势不变，融入世界开放发展的趋势不变。另一方面，国际金融危机的深层次影响依然存在，全球贸易增长乏力，宏观经济下行压力加大，我们面临着后发追赶、做大做强实体经济、做大做优经济“蛋糕”的发展考验；面临着投入大、产出小，收入少、支出多的运转困难；面临着发展失衡、民生欠账和经济社会风险隐患增多的严峻挑战。我们必须全力抢抓机遇，主动应对挑战，提振发展信心，提升发展能力，开创新常态下发展新局面。

　　“十三五”时期，我市经济社会发展的主要目标和任务，体现在“奔小康、建中心、增后劲、优生态、惠民生”。“奔小康”就是全面建成小康社会。到2018年，经济发展、民主法制、文化建设、人民生活、资源环境5个领域39项指标基本达标，全市小康综合指数达到97%以上，提前实现生产总值和居民人均可支配收入比2010年翻一番，力争迈进广东经济发展第二梯队。“建中心”就是加快建设环北部湾中心城市。环北部湾临港产业、交通枢纽、区域物流、金融商贸、科教创新、文化艺术、医疗卫生、旅游度假、康体运动和会展博览“十大中心”地位有效突显，先进制造业、港口物流业和基本公共服务三大优势全面提升。“增后劲”就是发展后劲进一步增强。主导产业、新兴产业、区域增长极培育发展和传统产业转型升级有重大突破；高铁、公路、机场、港口等重大基础设施建成运行，交通条件全面改善，中心城区、县城和中心镇公共服务功能全面提升。“优生态”就是生态环境进一步改善。争取完成“五城同创”、国家循环经济示范城市建设和生态文明区城镇村五年行动计划，单位GDP能耗水耗、二氧化碳以及主要污染物排放控制水平处在全省前列，空气质量位居全国重点城市前列。“惠民生”就是社会民生事业全面进步。科学、教育、文化、卫生、体育、妇女、儿童等民生事业加快发展，就业与社会保障体系健全完善，基本建立惠及城乡群众的公共服务体系。

　　实现“十三五”发展目标，我们必须坚持以经济建设为中心，坚持发展是硬道理的战略思想，坚持在创新、协调、绿色、开放、共享五大发展理念引领下，推动湛江发展迈上新台阶。我们要坚持创新引领，更大力度激活发展之源。全面实施创新驱动发展战略，推进供给侧结构性改革，鼓励大众创业、万众创新，优化投资、消费、出口结构，打造经济增长“新引擎”。我们要坚持协调平衡，更大力度补足湛江之短。着力推进陆海统筹，完善半岛立体交通，建设半岛港口群，发展临港工业，壮大海洋经济，实现蓝色崛起；着力推进城乡统筹，强化“一湾两岸”中心城区龙头引领，聚焦县城支撑节点，做强县域经济，形成区域城镇链。我们要坚持绿色低碳，更大力度呵护湛江之美。坚定走生产发展、生活富裕、生态良好的绿色发展之路，推动城市与产业双重转型，加快建设“三个城市”，构建资源节约型、环境友好型社会，永葆天蓝、地绿、水清。我们要坚持双向开放，更大力度实现湛江之变。坚定方向，发挥优势，深化合作，外接东盟、欧美，内联北部湾、珠三角，建设“一带一路”海上合作战略支点城市。我们要坚持共建共享，更大力度夯实民生之本。坚守底线、突出重点，完善制度、引导预期，有效保障和改善民生，推进城乡基本公共服务均等化，让全市人民共享全面小康成果。

　　三、2016年工作安排

　　2016年是“十三五”开局之年，也是推进结构性改革的攻坚之年。今年政府工作总的要求是：全面贯彻党的十八大、十八届三中、四中、五中全会和中央经济工作会议精神，深入学习贯彻习近平总书记系列重要讲话精神，认真贯彻省委十一届五次、六次全会和市委十届八次、九次全会精神，按照“五位一体”总体布局和“四个全面”战略布局，牢固树立和贯彻落实创新、协调、绿色、开放、共享的发展理念，围绕全面建成小康社会和加快建设环北部湾中心城市的总目标，紧扣结构深度调整、做大做强实体经济这一主线，适应经济发展新常态，坚持改革开放，坚持稳中求进工作总基调，推进供给侧结构性改革，推进陆海统筹、城乡协调和创新驱动，推进城市与产业转型发展，改善社会民生，建设生态文明，努力实现“十三五”良好开局。

　　今年经济社会发展的预期目标：生产总值增长10%左右；规上工业增加值增长12.5%；固定资产投资增长15%以上；港口吞吐量增长10%以上；社会消费品零售总额增长12%；居民消费价格指数涨幅控制在3%左右；外贸进出口总额增长10%，实际利用外资增长5%；公共财政预算收入增长8%；居民人均可支配收入增长8%；城镇登记失业率控制在3%以内；高中阶段教育毛入学率保持在90%以上；人口自然增长率控制在11.58‰以内；城镇生活垃圾无害化处理率85%。

　　做好今年政府工作，实现发展预期目标，最重要的是深入学习贯彻习近平总书记系列重要讲话精神，坚持把认识新常态、适应新常态、引领新常态作为贯穿发展全局和全过程的大逻辑，弄懂弄通“对新常态怎么看”、“新常态下怎么干”，彻底抛弃用旧的思维逻辑和方式方法再现高增长的想法，坚持用创新、协调、绿色、开放、共享五大理念引领发展实践，全面落实中央提出的“十个更加注重”要求，加快工作重点转变。具体抓好以下工作：

　　（一）综合施策优化发展环境，精准发力稳定经济增长。壮大实体经济。实施“去、降、补”行动方案，着力促进去产能、去库存、去杠杆、降成本、补短板。打好降低企业交易、人工、社保、财务、物流成本“组合拳”，清理规范中介服务，降低制度性交易成本；清理不合理收费，编制涉企收费清单目录，落实结构性减税优惠和制造业增值税税率降低政策，减轻企业税费负担；推进电价市场化和流通体制改革，降低企业用电与物流成本。平等对待各类市场主体，确保中央、省市改革政策落实到位，确保各类帮扶、奖励政策全面落实到民营企业，打破民间投资的“玻璃门”、“弹簧门”，努力营造公开透明的政策环境、高效务实的行政环境、平等竞争的市场环境，减少对民间资本的不合理限制，聚力推动民营经济大发展。采取兼并、债务重组或破产清算等方式，帮扶困难企业，妥善处置“僵尸企业”，促进优胜劣汰和市场出清。强化经济运行预警监测，加大价格监管。改进统计工作，为经济发展提供科学支撑。

　　突出创新驱动。坚持把创新驱动作为核心战略和总抓手，牢牢把握发展的主动权。建成“南方海谷”启动区一期工程，完成投资6.8亿元；加快霞山智慧产业城建设；坚持同步推介招商，落实中冶集团海水淡化等20多个入驻项目，建设创业生活、科技创新、产业集聚“三位一体”的复合型创新社区，打造“众创”新高地。支持广东海洋大学、广东医学院、岭南师范学院等驻湛高校创新发展。支持发展创客空间等新型孵化模式，打造众创、众包、众扶、众筹支撑平台。鼓励县（市、区）及社会力量发展众创空间和网络众创平台，支持企业采用众包模式聚合员工智慧和社会创意，提升设计研发、生产制造和运营维护水平。强化企业创新主体地位，实施高新技术企业培育工程，新增高新技术企业20家，鼓励有条件的企业设立研发机构。申报国家高新区。加快发展实物、股权众筹和网络借贷，用好科技风险投资引导和创业投资信贷风险补偿资金，强化知识产权保护，营造共助小微企业和创业者成长的社会氛围。牢牢抓住人才这个根本，建设北部湾人才高地。着力优化人才环境，加强和改进高层次人才、海外人才、高技能人才的引进培养及评选交流工作，切实解决引进人才落户、子女读书等实际困难。办好第二届“南方海谷杯”海洋科技创新创业和“市长杯”工业设计大赛。

　　强化有效投资。坚持在基础设施、农村水电气路、城市快速交通、地下管廊、保障性住房和生态保护、环境治理等公共领域加大投资力度。支持民营企业参与政府投资项目和重大基础设施项目，参与发展市政公用事业，支持民办学校、民办医院健康发展。推广PPP模式，实施海东新区地下综合管廊、奋勇高新区军民结合产业园基础设施等示范性项目。年内安排重点项目174项，开工47项，建成16项，完成投资349.3亿元。扎实推进“十百千投资计划”和“乡贤反哺工程”，确保签约项目快落地、快开工、快建成。注入有效资源，鼓励发行债券，增强基投、交投、水务和旅游集团投融资能力。

　　扩大新型消费。加快改造城市商圈，鼓励餐饮、零售、住宿等传统商业发展O2O模式，推行“网订店取”，促进线上线下消费融合，推动养老家政、健康信息、旅游休闲等生活性服务业开拓电子商务市场。加快发展住房租赁市场，鼓励农业转移人口在就业地买房或长期租房。鼓励自然人和各类机构投资者购买库存商品房，发展以住房租赁为主营业务的专业化企业。支持房地产企业实施库存商业、办公用房“商改住”，将库存商业地产改造为创客空间或商住复合式地产。优化公积金住房提取使用政策，增强职工购房能力。加快宽带提速降费，扩大信息消费，挖掘农村信息消费潜力。落实带薪休假，鼓励旅游消费。完善公共文化服务体系，提高文化消费水平。

　　稳定出口增长。实施优进优出战略，促进外贸结构调整、转型升级。支持企业扩大东盟市场，深耕欧美市场，拓展中东、非洲等新兴市场。用好出口信保等金融工具，培育发展外贸新业态。创建境外市场公共销售平台，发展便利跨境电子商务。完善电子口岸网络平台，实施单一窗口和通关一体化，申请设立海关多式联运监管中心；创建宝满国际卫生港和出口食品质量安全示范市，增强出口产品竞争力。

　　（二）推进供给侧结构性改革，加快构建现代产业体系。坚持在适度扩大总需求、调整需求结构的同时，突出供给侧结构性改革，着力提高产品、服务和工程质量，满足人民群众日益增长的品质、个性、绿色、体验和智能等多样化消费需求，实现低水平供需平衡向高水平供需平衡跃升。

　　着力发展临港工业。推进陆海统筹，坚持海洋有序开发与有效保护并举，依托现代港口壮大海洋产业，加快发展海洋经济。全面落实《中国制造2025》和省智能制造发展规划，培育发展精品钢材、海工装备、军工产业等先进制造业，建设海洋产业集聚区。全力保障钢铁基地１号高炉稳定达产，２号高炉9月前建成投产，全面建成钢铁一期工程，打造国内最具竞争力的精品钢基地。实施“钢铁带动工程”，发展钢铁配套产业，加快建设钢铁配套园区道路和污水处理等基础设施，加快实施海兴机械等30个入园项目，力争18个项目建成投产。加快德佬村搬迁，保障钢铁基地通过环保验收。尽早动工中科炼化主体工程。支持晨鸣纸业四期建成投产，启动第二轮投资。加快建设大唐雷州火电、京信东海电厂、鹏尊能源等重点项目，争取徐闻京能火电项目早日落户。实施工业强基工程，发展工业互联网，促进机器人应用，推动建设智能工厂、数字化车间，规划建设智能装备制造基地。支持建设广东普奥思无血清细胞培养基研发生产项目，壮大生物医药产业。落实传统支柱产业转型升级路线和行动计划，推进扩产增效、设备更新和智能化、绿色化改造，重点支持150家规上企业完成技术升级改造，规划建设糖业精深加工产业园，促进家电、家具产业园提质增效，建设羽绒、鞋业、再生资源产业基地，支持农海产品和食品加工业提升精深加工水平。

　　提升园区发展规模和质量。创新“三讲三评”机制，开展园区“三评三比”，推动项目、资金、用地和环境指标向园区倾斜，探索股权投资，用好省园区扩能增效资金，高标准建设园区基础设施和标准厂房。完善土地利用总体规划，优化用地布局，拓展用地空间。加快东海岛道路、排水工程和自来水厂建设，力促石化园区华南联合等７个项目落地建设。加快建设麻章森工产业园，完成5.5平方公里启动区征地拆迁。支持奋勇高新区打造军民融合、东盟合作、科技创新与产业集聚四大平台，形成食品医药和机械装备产业集群。加快建设乌石、龙头沙等县域临港产业园，规划建设吴川空港经济区和坡头先进制造产业园区。

　　加快发展现代服务业。办好中国海博会、水博会和湛江海洋周，举办东盟、海峡两岸现代农博会和南珠展览会，打造中国冬季会展品牌。创建广东服务外包示范城市和示范园区，建设粤港澳服务贸易自由化省级示范基地。推广“投递通”等智慧物流，发展第三方物流。建成湛江商贸物流城和义乌小商品批发城一期。一季度验收营运保税物流中心，建设华南铁矿石现货交易和检验鉴定中心，建成铁矿石、原油、燃料油期货交割库，打造中国南方大宗商品物流交易中心，壮大港口物流业。落实全域旅游观念，加快实施“3844”工程，完善城市旅游公共服务设施；推进建设“五岛一湾”滨海旅游产业园，建成法式风情街二期，加快建设南三岛滨海旅游示范区，推进硇洲岛旅游开发，创建湖光岩5A和金沙湾、特呈岛4A景区，打造湛江湾旅游圈。支持吴川吉兆湾打造滨海旅游集聚区，建设徐闻“中国大陆南极村”。

　　培育发展信息产业。落实信息基础设施建设三年行动计划，建设全国电信普遍服务试点市，推进“一网三环”光纤骨干网扩容，加快城市光纤改造，发展农村光纤网络，加快4G基站规模化建设，实现公共区域无线局域网覆盖。加快中国移动（湛江）数据中心项目建设，打造粤西高成长性信息产业集群。实施湛江“互联网+”行动计划，加快建设智慧城市，申建国家电子商务示范城市，推动“三网”和电商、网购、快递等行业融合发展，推广智能交通、电网、家居和智慧旅游、社保、医疗、食品溯源等物联网示范应用。支持建设赤坎跨境电子商务产业园，实施遂溪农村淘宝项目。

　　积极发展现代农业。全面落实中央一号文件，深化农村综合改革，稳步推进农村“三权”确权登记颁证，规范集体“三资”管理。严守耕地保护红线，推进建设高标准基本农田。鼓励发展农业适度规模经营，加快建设雷州东西洋现代粮食产业和廉江、徐闻国家现代农业示范区；加快木瓜、香蕉、红橙、青枣、火龙果等特色农产品产业化，建设雷州半岛热带水果产业示范区；提高蔬菜种植规模化水平，建设粤西“北运”蔬菜优势产区。完善农产品冷链仓储配送系统，推行家禽“集中屠宰、冷链配送、生鲜上市”。建设北部湾农产品流通综合示范园二期，建成湛江粮食物流中心。实施普惠金融村村通工程，普及乡村金融（保险）服务站；推广“政银保”、小额信用以及农村产权抵押担保贷款，推动水稻、甘蔗、香蕉、玉米等政策性农业保险扩面提质；开展台风、强降雨等财政巨灾风险指数保险试点，建立巨灾保险制度。加强雷琼黄牛、徐闻黑山羊等地方良种保护开发，发展现代畜牧业。发展现代渔业，建设全国重要远洋渔业基地。鼓励深海网箱养殖。积极拯救南珠产业。动工建设硇州渔港等6个省级现代渔港，建成龙王湾、通明河等5个市级区域性避风塘。完成两宗中型灌区节水改造和1宗规模化节水工程，完成12宗小型病险水库除险加固、15宗中小河流治理和27宗海堤加固达标工程。

　　（三）加快实施“三环四通”工程，着力提升发展区位优势。深入开展交通建设大会战。年内实施17个重点交通建设项目，完成投资90亿元。加快建设东海岛铁路和铁路西站客运综合交通枢纽工程，推进黎湛铁路电气化改造与合湛高铁建设，启动包海高铁玉林至湛江段、湛江至河源高铁前期工作。掀起高速公路建设高潮，加快建设东雷高速、云湛高速湛江段和湛徐高速徐闻港支线，动工建设玉湛高速湛江段和调顺跨海大桥，力争动工建设环城高速南三岛大桥段，启动廉江至湛江、吴川至湛江两条快速线前期工作，争取早日动工建设。启动雷湖快线奋勇延长线前期工作，开展海川路、疏港大道市政化改造，推动实施沈海高速开平至湛江段扩建和高速公路、铁路与县域港口连接线工程。改造省道285线吴川良村至梅北等7段道路，完成“迎国检”收尾工作，力争提前完成“3年改造300公里县道”目标。动工建设湛江国际机场。

　　加快建设半岛港口群。修编湛江港总体规划，加快推进湛江港40万吨级航道、亚士德航道一期工程，动工建设东海岛港区杂货码头，加快实施东海岛港区航道、煤炭码头、散货码头一期和宝满港区集装箱一期扩建工程，推进中科炼化、京信东海电厂两个配套码头建设。推行“政府+港口集团+园区业主”的县域港口建设模式，实施“一县一港区”战略，启动雷州乌石临港工业园货运码头、廉江龙头沙公共码头和徐闻南山滚装码头等县域港口工程。

　　（四）坚持以人为核心，提高城市建设管理水平。贯彻中央城市工作会议精神，落实城市工作“一个尊重”、“五个统筹”，开展城市扩容提质“三评三比”，提高城市治理能力，推动城市转型发展，创造更加优良的人居环境。

　　加强和改进城市管理。创新城市规划理念和方法，编制新型城镇化规划；加强城市设计，落实城市空间立体性、平面协调性、风貌整体性和文脉延续性的规划管控措施，留住城市特有的地域环境、文化特色和建筑风格基因。把2016年定为城市管理年，改革执法体制，落实城管责任主体，成立市城市管理委员会；坚持区级属地管理、市级监督考核、城管综合执法，坚持全民共治共管、共建共享，广泛发动市民参与，提高城市精细化管理水平。加大查控力度，整治城市“六乱”，打击市区违法建设。发挥数字化城管和交警智能指挥中心的平台作用，应用科技装备，实现城市管理全时段、全覆盖、全过程，做到城市管理问题第一时间发现、派遣、处置，确保城市运行高效有序。

　　推进新城区开发。持续释放省运会举办地的品牌效应，加快土地储备，聚力开发海东新区。加快地下综合管廊、污水处理厂、自来水厂和沿海大道等公共基础设施建设，加快实施广东医学院附属医院海东院区、渤海商品交易中心等重点项目。加快征地和村场改造，建成三柏西村农民公寓。坚持公共交通开发导向，加快建设海大路，动工建设西城快线，加快市政干道和公共服务向西延伸，做好西城组团规划实施工作。

　　推进主城区提质。年内安排市政项目19个、完成投资7亿元。建成广州湾大道南段、南方路排水及跨线桥、源珠路西段和世贸人行天桥，建设人民大道南地下步行系统。动工建设、改造文西路等10条市政道路，改造机畅路。综合整治霞山西海岸观海长廊，建成东菊公园。加快棚户区和危房改造，综合整治老旧住宅小区，加快旧大天然、三星、泰伦和调顺岛海洋渔业公司片区“三旧”改造。推进湛江港调顺、霞海港区功能腾退，推动湛化集团、海滨船厂、湛江电厂、调顺电厂环保迁建，力争开工建设湛化新厂。加快路灯安装亮化，打造人民大道南和观海长廊夜景示范街。加快城市原点广场二期、中央商务区和渔人码头建设。

　　推动县城和重点中心镇建设。坚持城镇、交通、产业、生态和动力“五链融合”，支持县城做大做强。编制县城控制性详细规划，突出建成一批市政交通设施，完善一批公共服务设施，完成一批“三旧”改造项目，建设一批科技企业孵化器。支持雷州、吴川、遂溪城区国道改道绕行、扩容提质；支持海安开发区功能调整，推动徐闻城区向南发展。推进建设廉江安铺等重点中心镇和特色村。

　　（五）加强生态环境保护，促进绿色低碳循环发展。扎实推进“三个城市”建设。按照“精明增长”、“紧凑城市”要求，实施脚印城市建设八大行动计划。动工建设广州湾大道北段自行车“高速公路”；建设赤坎民国风情街，改造逸仙路片区民国旧建筑群；建设西连接线综合公交枢纽站场；完善市区电动车充电设施。落实海绵城市建设六大行动计划，申报国家（省）海绵城市试点市，开展新建道路和河渠低影响开发雨水系统试点，实施海田带状公园等植草沟改造。落实国家循环经济示范城市建设任务，完善再生资源回收体系，推动餐厨废弃物等城乡生活垃圾资源化利用、无害化分类减量处理，推广绿色建筑和建筑节能，限制一次性产品消费，健全社会资源循环利用和节约标准体系。综合推进金沙湾片区“脚印”路网、“海绵”试点和“循环”社区建设，试点城乡生活垃圾分类回收和集中处理，打造城市转型发展样板片区。

　　加快实施主城区治水工程。落实主城区治水十年规划和三年计划，开展北桥河、南柳河、赤坎水库等河渠水库治理。建成东山垌、三号渠、文保渠生态廊道和瑞云湖、赤坎江生态处理系统。启动鹤地水库向市区管道供水工程前期工作，新建霞山西厅和东海岛自来水厂。加快改造地下排水系统，集中整治市区4个“水浸街”黑点。

　　筑牢生态安全屏障。坚持城市以自然为美，打造亲水近海空间，整治修复海岛、海岸带，建设美丽海湾和海洋生态文明示范区。实施现代农业、水利设施、生态修复三大规划，建设雷州半岛生态修复示范工程。扎实推进“五城同创”，编制实施低碳生态城市建设规划，提升生态文明区城镇村创建成效，提高农村卫生保洁员补助标准。开展城镇、农村地籍调查，建设国土资源“一张图”数据库；落实最严格的节约集约用地制度，加大闲置土地处置力度。扎实开展大气、水污染防治行动，落实粤桂两省区九洲江流域联防联治措施，坚决整治非法抽砂行为，争取鹤地水库列入国家江河湖泊生态保护试点。综合治理鉴江中下游和袂花江、小东江、三丫江污染。新（扩）建赤坎水质净化厂三期等6间污水处理厂，建设屋山污水处理厂、平乐再生水厂中水回用工程。推进工业污染源达标排放，规范企业排污行为。推行环境执法与刑事司法联动，严厉打击环境违法行为。推进新一轮绿化湛江大行动，实施两万亩桉树纯林改造和热带雨林示范工程，营建3万亩沿海防护林等生态公益林；建设15个森林、湿地公园和160条绿化美化村庄。提高建筑抗风设计标准和工程质量，专项整治建筑余泥、渣土堆（排）放和地质灾害易发场所等安全隐患，实施危险化学品和化工企业生产、仓储安全环保搬迁，加快建设安全城市。

　　（六）推进重点领域改革攻坚，进一步释放市场活力和社会创造力。推进简政放权、放管结合、优化服务，深化行政审批制度改革，继续取消和下放一批行政审批事项，全部取消非行政许可审批事项，落实事中事后“接管放”。建立政府部门权责清单制度，基本完成市、县两级政府工作部门以及依法承担行政职能事业单位的权力清单公布工作，推行“负面清单、一表共用、联合检查”。改革投资审批制度，落实企业投资项目清单管理制度，推行“多评合一”、并联审批，有效缩短重点项目前期工作周期。深化商事制度改革，推行电子营业执照和全程电子化登记，实行“一址多照”和“一照多址”，探索“智能”监管，推行随机抽查、告知承诺等监督方法。统筹推进户籍制度改革，实施居住证制度。全面实施不动产统一登记制度。深化金融改革，发展融资租赁和金融租赁，用好中小微企业“互保金”，引导金融资源优质服务中小微企业。强化预算执行管理考核，盘活财政存量资金，提高财政支出效率。分类推进国企改革，优化国有资本布局，盘活国有资产，鼓励各类投资主体参与国企改制重组，有序发展混合所有制经济，推动国企改制上市；建立出资人管理事项清单，健全国有资产管理制度，防止国有资产流失。深化海域使用权“直通车”制度改革，试点海域使用权有序流转和集中集约用海。深化供销社和国有林场体制改革。

　　（七）坚持内外联动，促进高水平双向开放。向南对接东盟加快开放发展。坚定对接东盟，全面提升与东盟等“一带一路”沿线国家和地区的合作发展水平。深化港口合作，推动落实21世纪海上丝绸之路沿线港口合作《湛江宣言》，参与共建港口和空港联盟，共建海上物流大通道、海上丝绸之路空中走廊和数字海上丝绸之路。举办“走进东盟”系列讲座，落实湛江与东盟14项合作事项，支持奋勇高新区建设“中泰”、“中印尼”东盟产业园，实施中泰合资制药等项目；邀请泰国、印尼参加2016中国海博会、水博会，设立中国海博会泰国主宾国展示馆，启动湛江与泰国互派50名交换生项目。深化与东盟的国际产能合作，带动优势产能“走出去”，支持蔗糖、饲料、农海产品加工和建筑、电气等企业走向东盟开展境外投资，带动成套设备出口。申报设立进口水果指定口岸，支持建设湛江-东盟热带农产品批发展示中心。加密、开通前往东盟国家的空中航班、集装箱班轮。建设“湛江-东盟旅游走廊”。整合利用驻外经贸代表处和国际友城等外事资源，扩大国际交流合作。

　　向北拓展促进区域经济一体化。主动对接珠三角，融入北部湾，推动发展湛茂阳及粤桂两广沿海经济带，加快建设湛江-北海粤桂北部湾经济合作区，规划建设环北部湾沿海一级公路，支持建设环北部湾检测公共技术服务平台，推进湛江与广西北部湾城市群一体化。

　　务实推进“三地”融合发展。巩固军政军民团结，军地共创国家和省级双拥模范城；发展人工岛礁基础材料，共建综合补给和奋勇高新区国家级军民结合产业示范基地，启动申报湛江国家军民融合创新示范区。深化校地教育、科研、文化、艺术、公益和志愿者服务合作，打造人才培养合作共同体；支持驻湛高校、科研院所参与“南方海谷”建设，搭建科研成果产业化平台。支持湛江农垦改革发展，推进垦地共建现代农业示范区广前核心区。

　　（八）改善社会民生，让人民拥有更多获得感。全力补齐社会民生短板。坚持精准扶贫、精准脱贫，瞄准建档立卡贫困人口，加大资金、政策、工作投入力度，打好脱贫攻坚战。坚持就业优先，创新小额担保贷款，落实租金、培训和社保补贴，打造互联网+创客空间，鼓励大中专学生创业就业。坚持立德树人，以创建教育现代化先进市为抓手，建设在线“云课堂”系统，改善办学条件；推进公办义务教育学校校长、教师交流轮岗，加强教师队伍建设；落实新建住宅小区学校配建与移交使用政策，新（扩）建市区6所以上公办中小学校。支持广东文理职业学院申报升格本科院校，建成湛江幼儿师范专科学校。繁荣文化艺术、新闻出版、广播影视事业，发展积极向上的网络文化。加强古村落保护。改革机关事业单位养老保险制度，提高城乡居民养老保险基础养老金标准，落实征地农民社保资金分配制度。完善医保信息审核系统，与省内和广西北部湾地区实现异地结算，丰富医保“湛江模式”；健全救助申请家庭经济核对机制，推动医疗保险与医疗救助衔接，提高医疗保障水平。实施卫生强市战略，将医疗卫生工作的着力点落到基层、落到农村，由市统筹解决县级人民医院、中医院和妇幼保健院改造资金，支持各县（市）按照三级医院标准，至少创建一间高水平医院；全面推进县镇医疗卫生服务管理一体化改革；提高乡镇卫生院、村卫生站标准化水平，改善基层医院就医环境和医护人员的工作生活条件。落实一对夫妇可生育两个孩子政策，完善计划生育服务管理。办好环雷州半岛骑行等社会体育活动，推动开展全民健身运动。支持海关、检验检疫、海事、边检在开放发展中发挥积极作用。推进市档案馆二期建设。抓好外事侨务、民族宗教、妇女儿童、海防打私、人防、气象、地震、方志等工作。

　　办成十件民生实事。在全面落实省十件民生实事基础上，开工建设保障房4825套，建成1500套；完成31716户茅草房改造验收；新增市区中小学学位1万个；新增市区医院病床1100张；建成银帆公园；投放新能源公交车320台、出租车300台，新增市区公交线路12条；建成绿塘河污水提升泵站；解决150万农村群众饮水安全问题；建成104条自然村污水处理站；解决市直国资系统困难企业退休人员基本医疗保险问题。

　　办好十大民心工程。启动市第一中医院迁建和市妇幼保健院新院建设；完成湛江中心人民医院整体搬迁；建成广东海洋大学寸金学院新校区一期，筹建湛江科技大学；筹建湛江一中新校区；动工建设市二技校一期；动工建设市文化中心；出台符合国标的电动自行车上牌管理办法；建设湛江、雷州救助物资储备仓库；动工改造人民大道、湖光路等10条市政供水管道；加强社区戒毒、社区康复工作和网格化管理。

　　创新社会治理。坚持政府治理和社会自我调节、居民自治良性互动，构建全民共建共享的社会治理格局。支持社会组织开展公益慈善、科技推广、城乡社区与志愿者服务，加强行业协会商会自律管理，引导社会组织规范健康发展。持续创建平安湛江，完善基层社会治理体制，健全第三方机构调处社会矛盾机制。以基础信息化为重点，完善社会治安立体化防控体系，加强反恐斗争，开展打击刑事犯罪“飓风2016”专项行动，重点打击“两抢一盗”、涉毒、涉电信诈骗和涉金融领域等犯罪活动。抓好“七五”普法教育。强化信访源头治理，完善网上信访制度。健全劳资纠纷预防处置体系。提升防灾应急处置、消防应急综合救援和通信设施抗灾能力，建设应急装备、物资、避护场所和基层应急综合服务站，建设“保底电网”和“应急供水”设施。健全食品安全风险及食源性疾病监测网络，全程保障群众食品药品安全。落实党政同责、一岗双责、失职追责，全方位加强安全生产。

　　（九）建设法治政府，全面提升施政水平。落实《法治政府建设实施纲要（2015-2020）》，加强和改进政府工作。坚持严格管理与关心爱护相结合，全体公务员要从严从实依法履职、优化服务，严格按照党的原则、纪律、规矩办事，严守党纪国法“高压线”。同时，坚持政治上激励、工作上支持、待遇上保障、心理上关怀，旗帜鲜明为呕心沥血做事、不谋私利的干部撑腰鼓劲，支持广大公务员积极作为、敢于担当。坚持依法行政，推进依法决策，完善政府内部决策规程，严格执行市委和市人大常委会讨论决定重大事项的相关规定，主动接受人大依法和政协民主监督，认真办理人大代表建议和政协委员提案。推进政府事权规范化、法律化，加强政府绩效管理。出台一批涉及城乡建设管理、环境保护、历史文化保护的政府规章。严格规范执法，加强行政复议和行政应诉工作。拓展市政府行政服务中心实体和网上办事大厅功能。加强政府智库建设，提高决策科学化水平。坚持阳光理政，主动回应社会关切，定期召开新闻发布会，完善在线访谈、网络问政、12345市民服务热线等公众参与平台，扩大“两微一端”等政务新媒体信息发布受众面，推动政务决策、执行、管理、服务和结果全过程公开。坚持实干施政，强化用结果说话，狠抓重大任务落实，突出重点项目落地，坚持重点工作天天排、月月算，半年一小结，全年算总账。强化工作部署督促检查，健全问责机制，做到有责必问、问责必严，打通政策落实“最后一公里”。坚持清廉从政，牢记从严修身、律己、用权，从实谋事、创业、做人，推进“三严三实”专题教育制度化，完善作风建设长效机制。坚持纪严于法、纪在法前，严格执行中国共产党《廉洁自律准则》和《纪律处分条例》，严守廉洁从政各项规定，完善监察、司法、审计联动的行政权力监督机制，自觉接受公众和媒体监督。严格落实党风廉政建设责任制，推进廉洁政府建设。

　　各位代表！大海孕育湛江，成就了千年古丝路与今日新海丝的扬帆起航；自然眷顾湛江，写就了近代广州湾与现代新港城的演变历程；时代呼唤湛江，造就了三十年改革开放与新世纪海洋强市的黄金机遇；人民期待湛江，铸就了我们战胜灾害困难、追求全面小康的意志担当！今天，我们已经站在“十三五”的发展新起点，让我们紧密团结在以习近平同志为总书记的党中央周围，在中共湛江市委的坚强领导下，咬定目标，坚定信心，攻坚克难，扎实苦干，共同用智慧和汗水，奋力夺取全面建成小康社会、建设环北部湾中心城市的伟大胜利！
